# The Core Proteome of Biofilm-Grown Clinical *Pseudomonas aeruginosa* Isolates

**DOI:** 10.3390/cells8101129

**Published:** 2019-09-23

**Authors:** Jelena Erdmann, Janne G. Thöming, Sarah Pohl, Andreas Pich, Christof Lenz, Susanne Häussler

**Affiliations:** 1 Institute for Molecular Bacteriology, TWINCORE GmbH, Centre for Experimental and Clinical Infection Research, a joint venture of the Hannover Medical School and the Helmholtz Centre for Infection Research, Hannover 30625, Germany; 2 Research Core Unit Proteomics and Institute of Toxicology, Hannover Medical School, Hannover 30625, Germany; 3 Department of Molecular Bacteriology, Helmholtz Center for Infection Research, Braunschweig 38124, Germany; 4 Institute of Clinical Chemistry, Bioanalytics, University Medical Center Göttingen, Göttingen 37075, Germany; christof.lenz@med.uni-goettingen.de; 5 Max Planck Institute for Biophysical Chemistry, Bioanalytical Mass Spectrometry, Göttingen 37077, Germany

**Keywords:** bacteria, DIA, mass spectrometry, microbiology, SWATH

## Abstract

Comparative genomics has greatly facilitated the identification of shared as well as unique features among individual cells or tissues, and thus offers the potential to find disease markers. While proteomics is recognized for its potential to generate quantitative maps of protein expression, comparative proteomics in bacteria has been largely restricted to the comparison of single cell lines or mutant strains. In this study, we used a data independent acquisition (DIA) technique, which enables global protein quantification of large sample cohorts, to record the proteome profiles of overall 27 whole genome sequenced and transcriptionally profiled clinical isolates of the opportunistic pathogen *Pseudomonas aeruginosa.* Analysis of the proteome profiles across the 27 clinical isolates grown under planktonic and biofilm growth conditions led to the identification of a core biofilm-associated protein profile. Furthermore, we found that protein-to-mRNA ratios between different *P. aeruginosa* strains are well correlated, indicating conserved patterns of post-transcriptional regulation. Uncovering core regulatory pathways, which drive biofilm formation and associated antibiotic tolerance in bacterial pathogens, promise to give clues to interactions between bacterial species and their environment and could provide useful targets for new clinical interventions to combat biofilm-associated infections.

## 1. Introduction

Bacteria either grow as free-swimming planktonic cells, or organized into biofilms [1,2]. Microbial biofilm communities are embedded in a thick, self-produced extracellular matrix and proliferate attached to abiotic or biotic surfaces [3,4,5]. Their formation represents a universal bacterial survival strategy, where the encapsulated bacteria are protected against a diverse array of environmental stressors [6,7]. Biofilms occur in natural environments, but there are also biofilm-associated infections of the eukaryotic host, which are notoriously difficult to treat [8,9,10]. These include infections of the lower respiratory tract in cystic fibrosis patients and those resulting from biofilm growth on implanted medical devices [11,12]. In biofilm-associated infections, bacteria show increased resistance against antimicrobial therapy and the attacks of the host immune system [13,14]. There are several studies that analyzed the proteome of biofilm-grown *P. aeruginosa* [15,16,17]. However, although it is recognized that bacteria present in biofilms behave differently from their planktonic counterparts [18,19], there is only a limited amount of studies that compare protein expression or mRNA abundance in planktonic and biofilm bacteria across multiple bacterial strains of the same species [20,21,22]. Similar to comparative genomics, comparative proteomics of multiple strains could uncover shared as well as unique features among the strains [23,24,25,26]. The identification of a common biofilm specific protein expression profile might reveal new insights into core regulatory pathways and adaptation strategies of bacterial biofilm growth on the species level. Furthermore, the detection of common responses of bacterial strains to a growth environment that drives tolerance towards environmental stressors might serve as a basis for the development of novel treatment strategies aiming at combating biofilm-associated infections [26].

In this study, we describe the proteome of 27 clinical isolates of the opportunistic human pathogen *Pseudomonas aeruginosa*. Protein expression profiles were recorded from the isolates cultured under planktonic as well as biofilm growth conditions using Sequential Window Acquisition of all Theoretical fragment ion spectra mass spectrometry (SWATH-MS) [27]. SWATH-MS has emerged as a key data independent acquisition (DIA) technique that enables global protein quantification of large sample cohorts. It outperforms standard shotgun approaches using data dependent acquisition (DDA) in terms of detectable peptides and associated proteins as well as measurement reproducibility [28,29]. We used SWATH-MS to quantify proteins in 108 samples (27 strains, two conditions, two biological replicates) which were analyzed in two technical replicates. Analysis of the resulting isolate- and lifestyle-dependent *P. aeruginosa* proteome profiles clearly differentiated the biofilm and planktonic physiological states. Furthermore, we defined a common core proteome under biofilm conditions despite significant fluctuations in the abundance of some proteins across the clinical isolates. This variance was independent of the genetic background of the individual clinical isolates, and independent of the planktonic proteome profile. Our study furthermore confirms previous observations that the protein-to-mRNA ratios between different *P. aeruginosa* strains are well correlated [30]. This suggests that the mechanisms regulating protein abundances downstream of transcription are conserved in the species *P. aeruginosa*. 

## 2. Materials and Methods

### 2.1. Bacterial Strains and Growth Conditions

27 *Pseudomonas aeruginosa* strains from different European origins and infection sites were cultured like described before [31]. In brief, precultures were grown with shaking (180 rpm) at 37 °C in 4 mL lysogeny broth (LB) medium. For planktonic condition main cultures of 10 mL LB were started from one preculture at optical density (OD)_600_ = 0.02 and stopped at OD_600_ = 2. For biofilm condition samples from the same precultures as planktonic samples were inoculated to 100 µL LB in 30 wells of a sterile half-area, 96-well µClear microtiter plate (Greiner Bio-One, Kremsmünster, Austria). The microtiter plate was sealed with an air-permeable BREATHseal cover foil (Greiner Bio-One). After 48 h growth at 37 °C in humidified atmosphere biofilm-growth was documented by fluorescence microscopy (data not shown) and wells of the same sample were pooled. Samples were washed three times with ice-cold phosphate buffered saline (PBS) followed each time by centrifugation and removal of the supernatant. All samples were taken in biological duplicates.

### 2.2. Sample Preparation and Proteome Analysis

Washed pellets were lysed in 800 µL lysis-buffer and sonicated as described previously [32]. After sonication cell debris was removed by centrifugation and proteins in the supernatant were precipitated bytrichloracetic acid(TCA)/Acetone as proposed elsewhere ([33], Protocol 5). In brief, proteins were reduced by dithiothreitol (DTT) and precipitated by TCA. The pellet was redissolvedin ice-cold acetone, spun down and acetone precipitation was repeated. The resulting pellet was dried and redissolved in 25 mM ammonium bicarbonate/0.1 M RapiGest (Waters, Milford, MA, USA)-solution. Afterwards, protein concentration was measured by DC-ProteinAssay (Bio-Rad, Hercules, CA, USA) and 50 µg protein was used for in-solution digest. After reduction by DTT followed by alkylation (IAA), proteins were digested by trypsin. Digestion reaction was stopped by adding TFA. After centrifugation and rejection of the pellet, peptides in the supernatant were dried by vacuum centrifugation.

For generation of a peptide library, equal amount aliquots from each sample were pooled to a total amount of 80 µg, and separated into eight fractions using a reversed phase spin column [34] (Pierce High pH Reversed-Phase Peptide Fractionation Kit, Thermo Fisher Scientific, Waltham, MA, USA.). Protein digests were analyzed on a nanoflow chromatography system (Eksigent nanoLC425) hyphenated to a hybrid triple quadrupole-TOF mass spectrometer (TripleTOF 5600+) equipped with a Nanospray III ion source (Ionspray Voltage 2400 V, Interface Heater Temperature 150 °C, Sheath Gas Setting 12) and controlled by Analyst TF 1.7.1 software build 1,163 (all AB Sciex). In brief, peptides were dissolved in loading buffer (2% acetonitrile, 0.1% formic acid in water) to a concentration of 0.3µg/µL. For each analysis, 1.5 µg of digested protein were enriched on a precolumn (0.18 mm ID × 20 mm, Symmetry C18, 5 µm, Waters) and separated on an analytical RP-C18 column (0.075 mm ID × 250 mm, HSS T3, 1.8 µm, Waters) using a 90 min linear gradient of 5–35% acetonitrile/0.1% formic acid (v:v) at 300 nl min-1.

Qualitative liquid chromatography – tandem mass spectrometry (LC-MS/MS) analysis was performed using a Top30 data-dependent acquisition method with an MS survey scan of *m/z* 350–1250 accumulated for 350 ms at a resolution of 30000 full width at half maximum (FWHM). MS/MS scans of *m/z* 180–1600 were accumulated for 100 ms at a resolution of 17500 FWHM and a precursor isolation width of 0.7 FWHM, resulting in a total cycle time of 2.9 s. Precursors above a threshold MS intensity of 125 cps with charge states 2+, 3+, and 4+ were selected for MS/MS, the dynamic exclusion time was set to 30 s. MS/MS activation was achieved by collision induced dissociation (CID), using nitrogen as a collision gas and the manufacturer’s default rolling collision energy settings. Four technical replicates per reversed phase fraction were analyzed to construct a spectral library.

For quantitative SWATH analysis, MS/MS data were acquired using 65 variable size windows [35] across the 400–1050 *m/z* range. Fragments were produced using rolling collision energy settings for charge state 2+, and fragments acquired over an *m/z* range of 350–1400 for 40 ms per segment. Including a 100ms survey scan this resulted in an overall cycle time of 2.75 s. Two replicate injections were acquired for each biological sample.

### 2.3. Data Processing

Protein identification was achieved using ProteinPilot Software version 5.0 build 4769 (AB Sciex) at “thorough” settings. Spectra from the combined qualitative analyses were searched against the *Pseudomonas* genome database [36] protein annotations of *Pseudomonas aeruginosa* strain UCBPP-PA14 (5892 entries, May 2019) augmented with a set of 52 known common laboratory contaminants to identify 2275 proteins at a False Discovery Rate (FDR) of 1%.

Spectral library generation and SWATH peak extraction were achieved in PeakView Software version 2.1 build 11041 (AB Sciex) using the SWATH quantitation microApp version 2.0 build 2003. The list of identified proteins was truncated to 1% FDR, and the corresponding Peptide-to-Sequence Matches (PSMs) were imported. Peptide sequences shared between different confidently identified proteins were omitted. Following initial ion chromatogram extraction, retention times were calibrated using endogenous high confidence peptides and linear regression. Finally, peak areas for up to the top10 peptides per protein (by identification confidence) were integrated using the top6 transitions per peptide (by signal intensity) at an FDR of 1 % [37] using a mass window of 75 ppm and a retention time window of 14 min around the expected values.These peptides were filtered for those that are present in the proteome sequences of all analyzed strains, their areas normalized by total area sums and finally summed to protein area values of 1806 proteins. Proteome sequences were generated from consensus genome sequences produced with the mpileup option of the SAMtoolspackage [38] after DNA sequencing of the samples as described in [39]. Protein coding sequences were extracted based on the reference annotation, and ambiguous nucleotides (“N”) were replaced with reference nucleotides to be able to obtain proteome sequences. This, as well as the translation of these DNA sequences, was done using Biopython’sBio.SeqIO module [40]. In an additional filtering step, those proteins were excluded from the analyses that differed significantly (student’s t-test, *p*-value < 0.5, log2 fold-change ≤-0.5 or ≥ 0.5) between two batches measured 5 month apart. Processed proteome data is available in Supplementary Table S1. 

The mass spectrometry proteomics data have been deposited to the ProteomeXchange Consortium via the PRIDE [41] partner repository with the dataset identifier PXD015073.

mRNAs were measured as described elsewhere [39,42] (planktonic samples) or as part of another project [Thöming et al., to be published] (biofilm samples) but with the same protocol. Sequences were then mapped to the *P. aeruginosa* PA14 genome using stampy [43] with default settings. Resulting read counts per gene (RPG) were normalized by DESeq [44] in R [45] as described elsewhere [46]. Processed transcriptome data is available in Supplementary Table S2.

All enrichment analyses were conducted with the R-package bc3net [47] and a *p*-value < 0.05 as cutoff for significant enriched functions. 

Transcriptome data is available at NCBI’s Gene Expression Omnibus: GSE134231 (biofilm transcriptomes), GSE123544 (planktonic transcriptomes).

Proteins were considered to be differentially regulated, if the log2 mean ratio of all replicates was ≤ −1 or ≥ 1 (two-fold change) and the students’ t-test *p*-value was ≤ 0.05. Differential expression of all quantified proteins is listed in Supplementary Table S3.

## 3. Results

### 3.1. SWATH-MS Characterizes Protein Expression Profiles in Multiple P. aeruginosaIsolates

For SWATH analysis, a spectral library was generated from a pool of 36 samples. Eighteen of the analyzed clinical *P. aeruginosa* isolates were grown under biofilm and planktonic growth conditions. Following protein extraction and tryptic digestion, aliquots were pooled and the resulting peptides separated into eightfractions by means of reversed phase separation at basic pH. Fractions were analyzed in triplicate by data dependent acquisition mass spectrometry, and the combined spectra were searched against the *P. aeruginosa* reference strain UCBPP-PA14 proteome (containing 5893 protein sequences). This lead to the identification of 2275 proteins at a 1% False Discovery Rate, which was slightly more than what was found in a comparable study [48]. The resulting spectral library was used to detect peptides in 108 different samples (27 strains, two conditions, two biological replicates) analyzed in duplicate by SWATH-MS. This enabled quantification of 6232 peptides corresponding to 1996 proteins. To ensure reliable quantification across all isolates, the resulting peptide list was filtered to those present in all strains analyzed. Protein sequences of the clinical isolates were generated by mapping sequenced genomes against the *P. aeruginosa* PA14 genome and translating the resulting protein coding sequences. 5165 peptides (82.9%), representing 1806 proteins, were present in all strains analyzed. Further thorough filtering for batch effects resulted in a high-quality quantitation matrix of 1021 proteins consistently quantified across all isolates under both conditions. Sample preparation, acquisition strategy, instruments, and parameters were similar to Losensky et al. [49].

### 3.2. Proteome Expression Patterns Differ Between Physiological States

**Figure 1:**
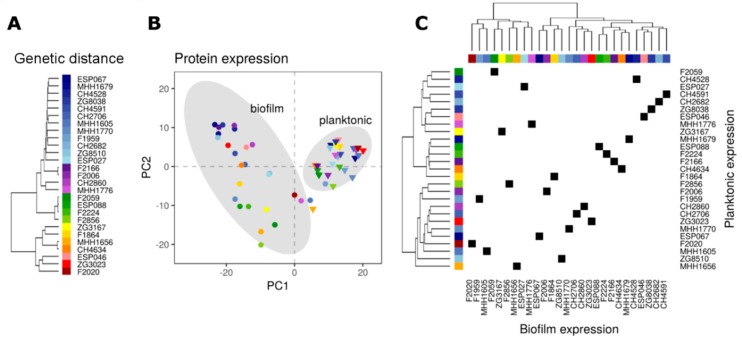
Protein expression profiles of biofilm and planktonically grown clinical *P. aeruginosa* isolates of diverse genetic background. (**A**) Hierarchical clustering by genetic distance based on the 1021 genes for which the encoded proteins were quantified in this study. PAO1-like strains predominate and are colored in blue-, violet-, and green colors, respectively. PA14-like strains are colored in yellow/orange or red. (**B**) Principal component analysis (PCA) of the isolates based on the acquired proteomic data. Biofilm (circles) and planktonic (triangles) proteomes of the clinical isolates (same color code as in A) are clearly separated. The grey areas represent the 95% confidence ellipse of the biofilm and planktonic proteomes, respectively. (**C**) Correlation matrix comparing the biofilm and the planktonic proteomic profiles of the clinical isolates. Each square reflects the position of an individual isolate on the two axes. The horizontal dimension represents the hierarchical clustering of protein expression data based on normalized expression of the 1021 proteins in the data set of clinical isolates grown under biofilm conditions, while the vertical dimension corresponds to hierarchical clustering of planktonic protein expression data. The phylogenetic strain background is indicated by colored subtrees (color code as in A) within the dendrogram.

From our collection of 414 well characterized clinical *P. aeruginosa* isolates [42,50] we selected 27, which represented the broad genetic distribution of the clinical strains (Figure 1A). These 27 isolates were cultured under planktonic conditions (until OD_600_ of 2) as well as under biofilm conditions for 48 h. Their protein expression profiles were recorded in two biological and two technical (MS measurement) replicates. We were able to consistently quantify 1021 proteins across all strains and conditions. 

Biofilm and planktonic proteomes could be clearly separated by principal component analysis (PCA) (Figure 1B), indicating that there were profound differences in protein abundances under these conditions, which were shared by the isolates. Although the 27 clinical *P. aeruginosa*isolates originated from diverse genetic backgrounds, they presented very similar proteomic profiles under planktonic conditions. In contrast, the biofilm proteome profiles were much more divergent between the strains. We also found that two isolates that clustered more closely under planktonic growth conditions did not necessarily cluster to the same extent, if grown in biofilms and vice versa (Figure 1C). Furthermore, there was no correlation between phylogenetic relatedness and the production of specific proteomic profiles, indicating that strains from any genetic background can produce distinct environment-driven proteome profiles. In conclusion, the 27 clinical isolates produced a higher proteome diversity when cultivated under biofilm conditions and this divergent protein profile was independent on the genetic background of the isolates and was not linked to the protein profile that was produced under planktonic growth conditions.

**Figure 2:**
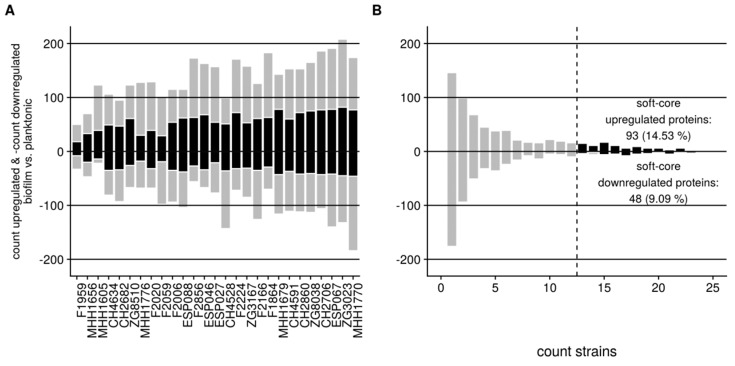
Differential protein expression between biofilm and planktonic growth conditions across 27 clinical isolates. (**A**) Number of proteins, which are up- and down-regulated in biofilm as compared to planktonic growth conditions, are shown for each strain. The number of proteins that belong to the set of soft-core biofilm proteins are colored black. Soft-core biofilm proteins are defined as those proteins, which exhibit an average expression level difference in all clinical biofilm grown isolates as compared to planktonic cultures and are found differentially expressed in at least 50% of the isolates. (**B**) Number of proteins that are commonly found differentially expressed in 1,2,3…27 isolates. No protein was found to be regulated in all of the analyzed (27) strains. The number of proteins that belong to the set of soft-core biofilm proteins are highlighted in black.

We next identified all proteins that were differentially expressed under biofilm versus planktonic growth conditions in each of the 27 clinical isolates (Figure 2A). Overall, 941 (92% of those quantified) proteins were differentially expressed at significant levels in at least one of the clinical isolates (Supplementary Table S3). The number of differentially expressed proteins between the two conditions ranged from 83 to 358 per isolate (Figure 2A). The great majority of proteins were found to be differentially regulated in only 1 to 6 of the 27 clinical isolates (Figure 2B). 

**Figure A1:**
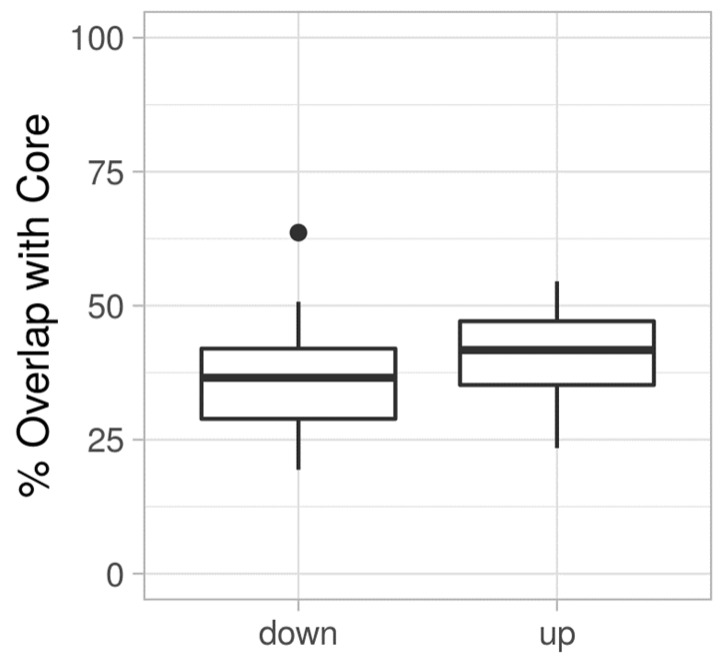
Overlap with core biofilm proteome for each strain.Percentage of significantly regulated proteins between a biofilm and planktonic condition that are also part of the core biofilm proteome. Data is divided into up-regulated (up) and down-regulated (down) proteins.

141 of the 941 proteins were differentially regulated (93 up-regulated, 48 down-regulated) under biofilm growth conditions in at least 50% of the isolates and were defined as ‘soft-core biofilm proteins’ (Supplementary Table S3). The relative percentage of these proteins among the differentially expressed proteins in biofilm versus planktonic growth had a median in the individual isolates of 41.7% within the up-regulated, and 36.6% within the down-regulated proteins, respectively (Figure 2A, Figure A1). 

Although environmental conditions were different from our study, we found an overlap with 48 h-biofilm-specific proteins in PAO1 published by Park et al. [15]. Out of the nine proteins that were also quantified in our study, eight were significantly up-regulated in biofilm compared to planktonic conditions. The five proteins that are also in our soft-core biofilm proteins because they had a more than two-fold difference in protein expression are:PdhB (PA14_19910), PA14_13140, the probable nonribosomal peptide synthetase PA14_11140, PA14_71240 and two-component response regulator PA14_30830.

**Figure 3:**
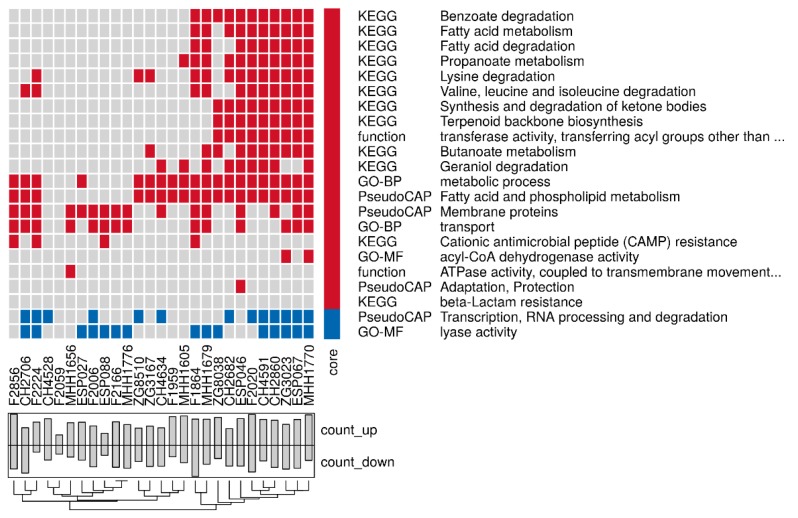
Enrichment of functional categories in the soft-core biofilm protein profile.All functional categories that were enriched (*p*-value < 0.05) in the group of proteins with a differential expression under biofilm as compared to planktonic growth conditions in at least 50% of the clinical isolates are listed. Function that were enriched in proteins that were up-regulated under biofilm condition in the individual isolates (x-axes) are colored in red, functions that were down-regulated are in blue. Bar plots indicate how many proteins were up and down-regulated in the individual strains. Functions were derived from KEGG-, PseudoCAP- or gene ontology (GO)-annotations. GO-MF = molecular function, GO-BP = biological process, GO-CC = cellular component.

Further analyses of the soft-core biofilm proteins revealed that despite substantial variability between the strains, we found a general increase in proteins involved in iron sequestration and iron metabolic processes (PchDG, FptA), in Pseudomonas quinolone signal (PQS) signaling (PqsBCDH), phenazine biosynthesis (PhzB1, PhzB2, PhzE2, PhzF2, PhzM), outer membrane proteins (OprCFG, MexI), and fatty acid biosynthesis (FabG). On the other hand we found a general down-regulation of proteins involved in translation processes in the biofilm-grown isolates (RplACQSX, RpmAG, RpsNRT, TruB3). Figure 3 depicts the enrichment of GO-, KEGG-, or PseudoCAP-annotations within the soft-core biofilm profile. Here, especially the PseudoCAP category “Fatty acid and phospholipid metabolism” was enriched in most strains under biofilm conditions. Other functional groups like the PseudoCAP category “Adaption, Protection” were enriched in the soft-core biofilm proteins but reached significance levels in only a few strains under biofilm growth conditions.

### 3.3. Proteome Expression Patterns Differ Between Groups of Clinical Isolates

**Figure 4:**
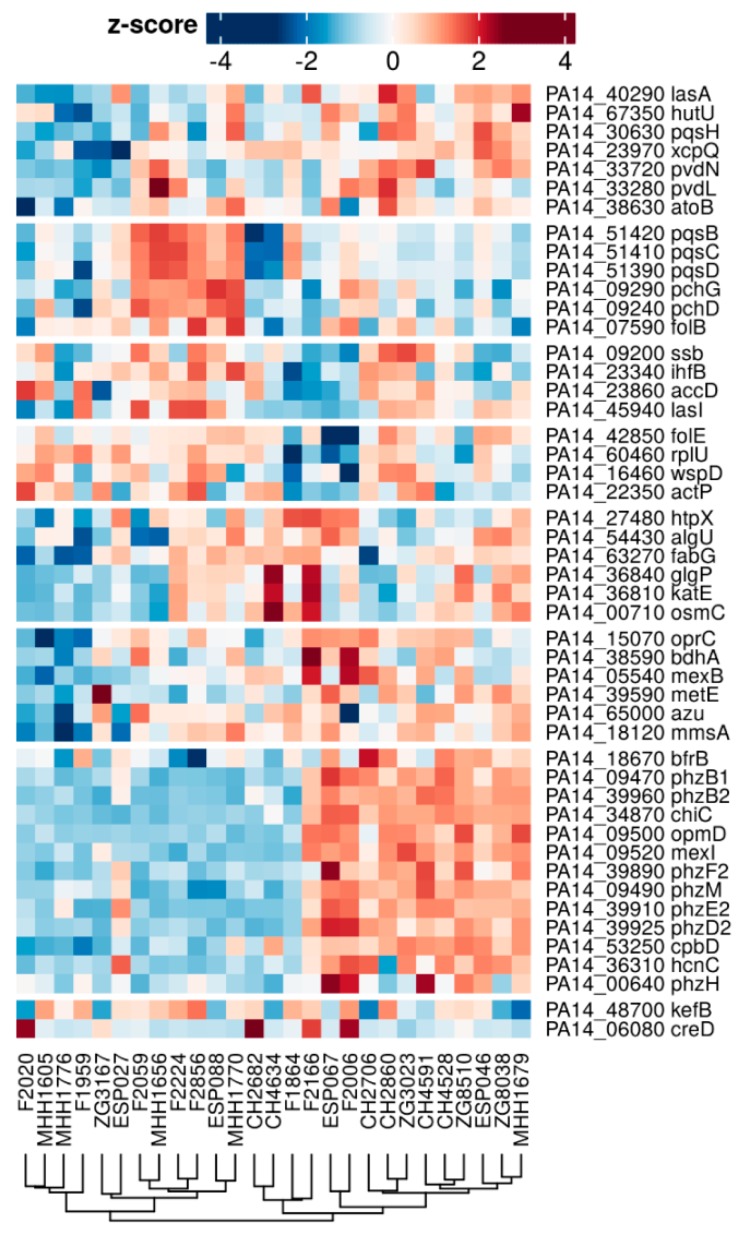
Distribution of expression patterns for variable proteins under biofilm condition.Out of the 10% most variable expressed proteins under biofilm conditions, 48 are annotated and listed here. The full list of variably expressed genes—also for planktonic conditions—is presented in Appendix Figure A2B,C.

**Figure A2:**
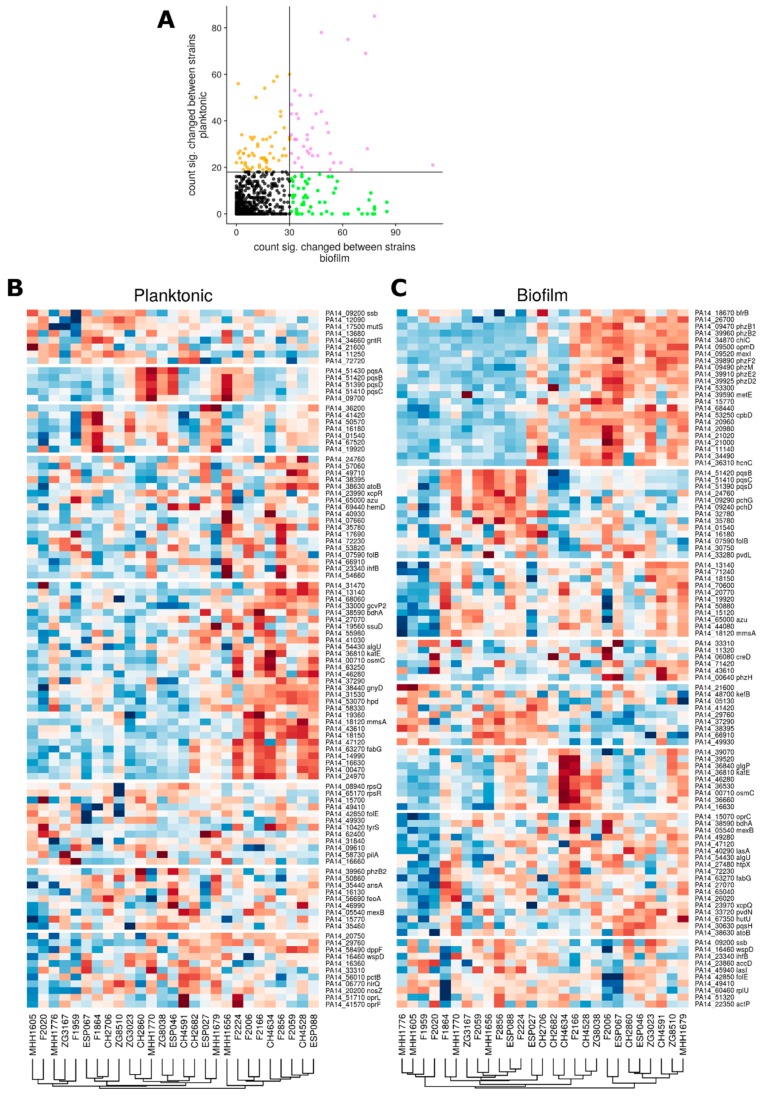
Variable protein expression between strains. (**A**) Comparison of variably expressed proteins under biofilm and planktonic condition. The count of significant differential expression between two strains is plotted for both conditions. The 10% most variable expressed proteins are shown in A and B. (**B**) Heatmap of 10% most variable proteins under planktonic condition. (**C**) Heatmap of 10% most variable proteins under biofilm condition.

In order to evaluate the variability of protein expression across the isolates when *P. aeruginosa* strains were cultured under the same environmental condition, we analyzed the 10% most variably expressed proteins within biofilm and planktonic growth conditions. 40% of the highly variable proteins overlapped under biofilm and planktonic conditions (Figure A2), indicating that those proteins were variably expressed between strains independent of the environmental conditions. Figure 4 shows a heatmap of the 48 most variably expressed and annotated proteins under biofilm conditions. Despite their variant expression across the 27 isolates, it seemed that there were sub-groups of isolates, which expressed groups of proteins at comparable levels. For instance, six isolates with various phylogenetic background expressed high levels of PqsBSD and PchDG compared to the other isolates, whereas eleven isolates expressed high levels of seven Phz proteins, as well as OpmD and MexI. 

### 3.4. Comparison of the Proteome and Transcriptome Biofilm Profiles

**Figure 5:**
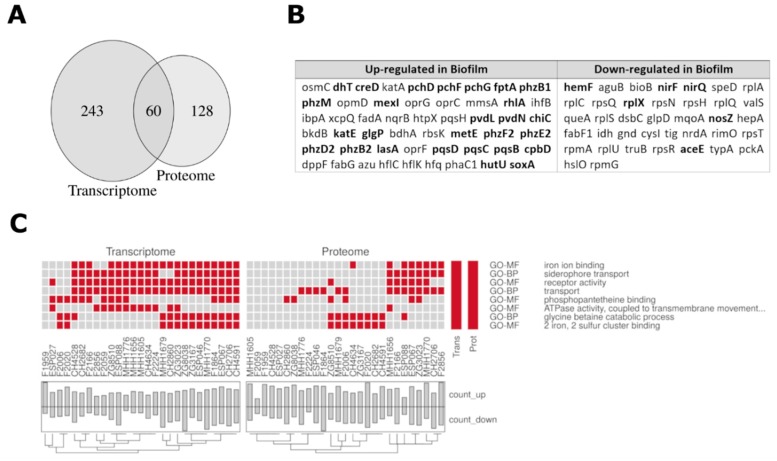
Comparison of differentially expressed proteins/transcripts in biofilm versus planktonic growth conditions. (**A**) Venn diagram showing the overlap of differentially regulated proteins and transcripts under biofilm versus planktonic growth conditions across the clinical isolates. Analysis of the differentially expressed transcripts was restricted to the 1021 mRNAs for which corresponding proteins were quantified in this study. (**B**) List of all annotated differentially regulated proteins throughout all strains. Gene names are printed in bold if the corresponding transcript was significantly regulated as well. Most of the overlapping proteins/mRNAs were upregulated under biofilm condition (49 out of 60). Out of these, 27 were annotated and listed here. From the remaining 11 proteins/mRNA that were higher expressed under planktonic conditions, six were annotated and listed. On the other hand, several annotated proteins were regulated in the proteome but not in the transcriptome. The complete list can be found in Supplementary Table S3. (**C**) All functional categories that were enriched (*p*-value < 0.05) in the group of proteins/transcripts with a differential expression under biofilm as compared to planktonic growth conditions are listed. The function that was enriched in proteins that were up-regulated under biofilm condition in the individual isolates (x-axes) are colored in red. Barplots indicate how many proteins are up- and down-regulated in the individual strains. Functions were derived from gene ontology (GO)-annotations. GO-MF = molecular function, GO-BP = biological process.

In the context of a further study [42] [and Thöming et al., in preparation] transcriptional profiles have been recorded for clinical isolates in biofilm and planktonic states. 303 genes were differentially regulated under biofilm versus planktonic growth conditions, if the 27 clinical isolates used in this study were considered as replicates. We found that the corresponding genes of almost a third of the differentially regulated proteins under biofilm conditions exhibited differential gene expression (Figure 5A,B, and Supplementary Table S3). This indicates that clinical isolates adopted distinct biofilm protein expression profiles that are shared among different clinical isolates, but that can only partly be deduced from the transcriptional profile under the same environmental conditions.

**Figure A3:**
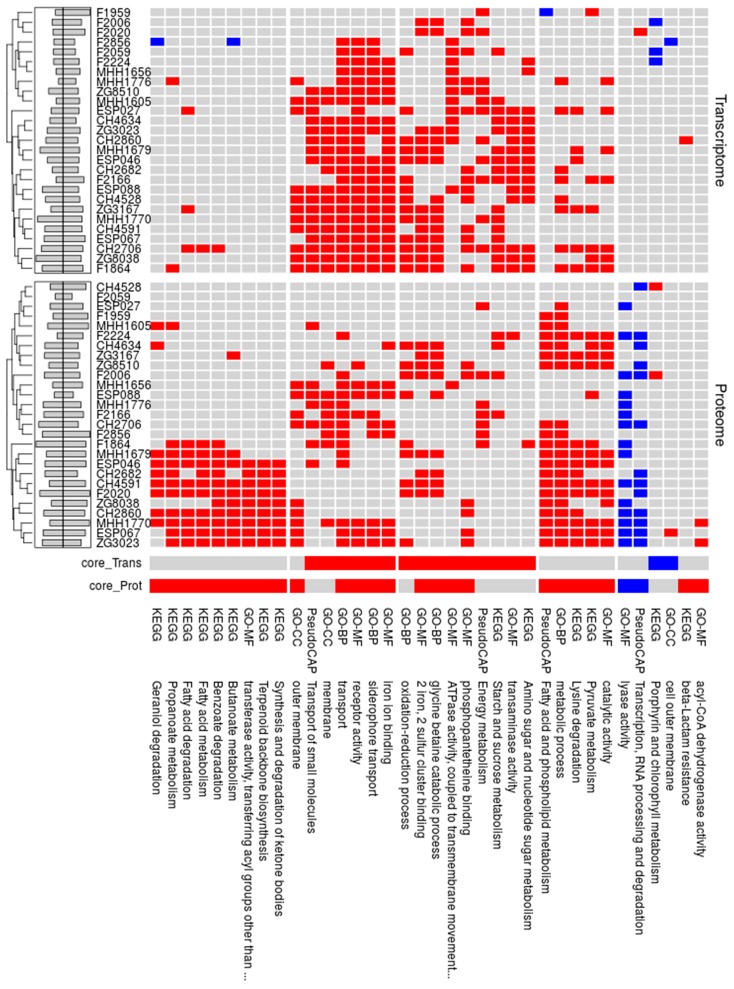
Functional enrichment biofilm/planktonic in protein and mRNA.All functional categories that were enriched (*p*-value < 0.05) in the group of proteins or transcripts with a differential expression under biofilm as compared to planktonic growth conditions are listed. The function that is enriched in proteins that are up-regulated under biofilm condition in the individual isolates (x-axes) is colored in red and down-regulated in blue. Barplots indicate how many proteins are up- and down-regulated in the individual strains. Core_Trans and core_Prot represent all isolates seen as biological replicates. Functions are derived from KEGG- (1), PseudoCAP- (2) or gene ontology(GO)- (3) annotations. GO-MF = molecular function, GO-BP = biological process, GO-CC= cellular component.

Figure 5B,C depict the genes/proteins and the corresponding functional groups, which were enriched in the proteome and transcriptome biofilm profiles across the 27 isolates (Figure A3). Especially iron-related and transport-functions were found.

**Figure 6:**
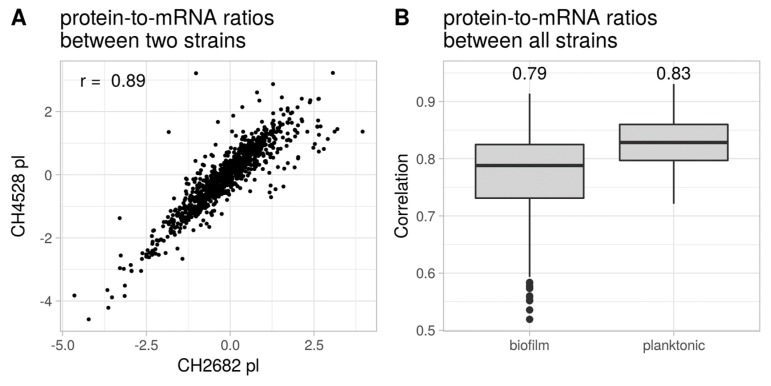
Protein-to-mRNA ratios across clinical *P. aeruginosa* isolates. (**A**) Exemplary plot of protein-to-mRNA correlation between two strains under planktonic condition. r = Pearson correlation (**B**) Median and interquartile range of the pair-wise correlation coefficients of all protein-to-mRNA ratios of the 27 strains under biofilm and planktonic conditions. Values on top of the boxplots represent the corresponding median.

Kwon et al. [30] previously recorded proteome and transcriptome profiles of the two *P. aeruginosa* type strains PA14 and PAO1 under exponential growth condition, and showed that protein-to-mRNA ratios were conserved between the two strains. Here, we could confirm their results. Figure 6A exemplarily shows a strong correlation (r = 0.89, Pearson) of the protein-to-mRNA ratios of overall 1021 gene/protein pairs exemplarily for two of the 27 clinical isolates. The median correlation coefficient of the pair-wise protein-to-mRNA ratios among all strains was in the same range under planktonic conditions (r=0.83), and slightly lower (0.79) under biofilm conditions (Figure 6B). Our results demonstrate that there are discrepancies between measured protein and transcript levels, which are however conserved across different *P. aeruginosa* isolates. This suggests that the respective transcripts might be targets of post-transcriptional regulation. Table A1 lists genes that exhibited a conserved higher or lower protein production than expected from the mRNA levels under biofilm and/or planktonic conditions.

## 4. Discussion

Comparative genomic approaches have facilitated the discovery of many previously unidentified conserved and species-specific regulatory pathways that determine phenotypic bacterial traits. In contrast to comparative genomic approaches, most of the proteome studies have been restricted to comparative analysis between two or few isolates or mutant strains. However, there have been significant technological and methodological advances in the recording of proteomes recently [49,51]. Proteomic studies that give information on protein expression profiles across a multitude of strains within one species have become feasible [21,52,53]. 

Using SWATH-MS, we identified differential expression of overall 1021 proteins across 27 clinical *P. aeruginosa* isolates. This corresponds to 17.3% of the 5893 predicted PA14 proteins, and thus compares well to the previously reported protein detection rates [21,52,54]. Despite the less-than-complete coverage of the entire proteome, the recording of extensive proteome profiles across genetically diverse clinical *P. aeruginosa* isolates promises to add a valuable layer of information and to expand genomic and transcriptomic data. Inter-disciplinary data integration strategies coupled with bioinformatics and biostatistics are promising to support a better understanding of biological systems and might open up new avenues for novel treatment strategies to combat bacterial infections. 

A previous study on the transcriptional profiles of biofilm-grown bacteria showed that there was only a relatively small number of common biofilm-specific endpoint transcripts in *P. aeruginosa* biofilms grown under different environmental conditions [55]. It was suggested that biofilm formation is a response to the environment, rather than a developmental pathway [56]. In this study, instead of growing one *P. aeruginosa* strain under various biofilm-promoting conditions, we grew various strains from different genetic backgrounds under one environmental biofilm condition. Interestingly, while the variability of the protein abundances across the different clinical *P. aeruginosa* isolates were low under planktonic growth conditions, the variability was substantially higher under biofilm growth conditions. Thus, despite the fact that the isolates were grown under identical biofilm promoting conditions, the differential expression of the biofilm protein profile was still variable across the clinical isolates. This may indicate that the genetic variation across the clinical isolates impacts to a greater extent on the biofilm profile, while the impact on the planktonic profile is lower. Clearly, further work on the correlation of genomic sequence variations with transcriptional and proteomic profiles is required in order to link different evolved mutations in clinical isolates to phenotypic bacterial behavior. 

Nevertheless, despite substantial variability, our analysis of the comprehensive proteomic dataset of 27 clinical *P. aeruginosa* isolates revealed common *P. aeruginosa* proteome changes that could be observed across multiple clinical isolates during growth within biofilms. For instance, among the 141 proteins of the soft core biofilm profile, we found a constant increase in proteins involved in iron sequestration and iron metabolic processes, in PQS signaling, outer membrane proteins and fatty acid biosynthesis. On the other hand, we found a general down-regulation of proteins involved in translation processes. A recent study, which compared in vivo *P. aeruginosa* proteome profiles across 11 cystic fibrosis (CF) patients with ex vivogrown *P. aeruginosa* populations from the same patient, identified a core set of differentially regulated proteins on the same order of magnitude (67 in vivo up-regulated and 117 down-regulated *P. aeruginosa* proteins) [21]. Although the conditions within the CF lung cannot be compared to the in vitro biofilm growth conditions of this study, the in vivo study also found an up-regulation of iron acquisition systems, and of outer membrane proteins and a downregulation of proteins involved in translation [21]. Regulation of these proteins might be a common theme due to biofilm-associated growth of *P. aeruginosa* in the lungs of chronically infected CF patients. 

In conclusion, while generalizing from one strain to a whole species might be problematic, the analysis of multiple isolates from one species has the potential to uncover important common adaptation traits that could become important targets, e.g., in the fight against biofilm infections. However, our study also demonstrates that there is a lot of variation across different clinical isolates and there does not seem to be a universal biofilm target. This indicates that an anti-biofilm strategy will have to consider more than one target in order to be successful against a broad range of different *P. aeruginosa* isolates. Furthermore, new information on proteomic changes can help finding undetected links between the genotype and the phenotype, especially if also genomic and transcriptomic data are integrated [57]. For instance, in accordance with a previous study [30], we demonstrated that there is a conserved protein-to-mRNA ratio across our clinical isolates even under different environmental conditions. This indicates that there may be a constitutive post-transcriptional regulation of defined sub-groups of genes in the opportunistic pathogen *P. aeruginosa*, whose importance for the adaptability of bacteria warrants to be further explored. 

## Supplementary Materials

The following are available online at https://www.mdpi.com/2073-4409/8/10/1129/s1, Table S1: Table_S1_proteome_data.xlsx, Table S2: Table_S2_transcriptome_data.xlsx, Table S3: Table_S3_differential_expression.xlsx.

## Appendix A

**Table A1:** Genes with the highest and lowest protein-to-mRNA ratio.The median of the 10 highest and lowest protein-to-mRNA ratio (log2) throughout all strains under the given condition.

Protein/mRNA (median)	Condition	PA14_ID	Gene.Name	Product.Name
3.06	pl	PA14_21010		FAD-dependent monooxygenase
2.67	pl	PA14_17930	glpD	glycerol-3-phosphate dehydrogenase
2.64	pl	PA14_34840		non-ribosomal peptide synthetase
2.63	pl	PA14_21020		non-ribosomal peptide synthetase
2.60	bf	PA14_35790		homospermidine synthase
2.41	pl	PA14_33280	pvdL	peptide synthase
2.38	pl	PA14_09280	pchF	pyochelinsynthetase
2.36	pl	PA14_00640	phzH	potential phenazine-modifying enzyme
2.29	pl	PA14_27370		ATP-dependent RNA helicase
2.26	bf	PA14_15580		Type II restriction enzyme, methylase subunit
−2.96	bf	PA14_15980	rimM	16S rRNA-processing protein RimM
−2.99	pl	PA14_15980	rimM	16S rRNA-processing protein RimM
−3.08	bf	PA14_18670	bfrB	bacterioferritin
−3.12	pl	PA14_64520		bacterioferritin
−3.15	pl	PA14_24770		hypothetical protein
−3.26	pl	PA14_68260		c4-dicarboxylate-binding protein
−3.53	pl	PA14_54430	algU	RNA polymerase sigma factor AlgU
−3.64	pl	PA14_18670	bfrB	bacterioferritin
−3.65	pl	PA14_23340	ihfB	integration host factor subunit beta
−4.22	pl	PA14_65000	azu	azurin
